# 

**Published:** 1888-06

**Authors:** 


### CONTENTS.

***MISCELLANEOUS.***

| | PAGE |
|---|---|
| What is a Proving? P. P. Wells, M. D., | 277 |
| Sartor Resartus. M. W. Vandenburg, M. D., | 278 |
| Lecture upon Podophyllum. Professor J. T. Kent, | 279 |
| Organon Society of Boston, Regular Meeting. S. A. Kimball, M. D., *Secretary*, | 283 |
| A Case of Retinitis Pigmentosa. Fred. W. Payne, M. D., | 286 |
| The Hahnemann Club of Toronto. J. D. Tyrrell, M. D., *Secretary*, | 291 |
| Post-Partum and Other Hemorrhages. A. B. Eadie, M. D. | |
| Platina. A. B. Eadie, | 293 |
| Sulphuric Acid. A. B. Eadie, | 294 |
| Stramonium. A. B. Eadie, | 294 |
| Chamomilla. J. D. Tyrrell, M. D., | 295 |
| Apis Mel. J. D. Tyrrell, M. D., | 296 |
| Arnica Mont. W. J. Hunter Emory M. D., | 296 |
| Apocyn. Cann. W. J. Hunter Emory, M. D., | 296 |
| Bryonia. W. J. Hunter Emory, M. D., | 297 |
| Hyoscyamus. Edward Adams, M. D., | 297 |
| Helonias Dio. Edward Adams, M. D., | 297 |
| Hamamelis. Edward Adams, M. D., | 298 |
| Aconitum. Hamilton Evans, M. D., | 298 |
| Caulophyllum. Hamilton Evans, M. D., | 299 |
| Erigeria. Hamilton Evans, M. D., | 299 |
| Proceedings of New York Homœopathic Union. Clarence W. Butler, M. D., *Secretary*, | 299 |
| Texas Homœopathic Medical Society, | 304 |
| Clinical Verifications. H. C. Morrow, M. D., | 305 |
| A Few Cases Terminating Successfully under the Higher Potencies. J. B. Garrison, M. D., | 307 |
| Rhus Rad. *vs.* Rhus Tox. Robert Farley, M. D., | 313 |
| A Case of Chronic Sulphur Poisoning. S. L., | 314 |
| A Case of Suppressed Gonorrhœa. E. W. Berridge, M. D., | 315 |
| Croton Tiglium. J. H. Hamer, M. D., | 320 |
| Class-Room Talks No. 5. S. L. G. L., | 324 |
| Contradictory Symptoms, | 327 |
| A Clinical Note,. | 328 |
| Clinical Cases. Stuart Close, M. D., | 329 |
| A Note on Theridion. Dr. Baruch, | 331 |
| ***BOOK NOTICES AND REVIEWS.*** | |
| Fifty Reasons for Being a Homœopath. J. Compton Burnett, M. D. London: The Homœopathic Publishing Co., 1888, | 331 |
| Pathogenetic and Clinical Repertory of the Most Prominent Symptoms of the Head. C. Neidhard, M. D. Philadelphia: F. E. Bœricke, 1888, | 331 |
| A Repertory of Gonorrhœa. Samuel A. Kimball, M. D. Boston: Otis Clapp & Son, 1888, | 332 |
| Odium Medicum and Homœopathy, | 332 |
| Fox's Atlas and Text-Book of Skin Diseases. E. B. Treat & Co., New York, | 332 |
| Lomb's Prize Essays. American Public Health Association, | 333 |
| New Jersey State Homœopathic Medical Society, | 333 |
| Homœopathic League Tracts No. 18, | 333 |
| ***NOTES AND NOTICES.*** | |
| I. H. A.—Removals—Ohio Homœopaths—New York Homœopathic Medical College—Lachesis—Clinical Work—Testimony for High Potencies—Mental Symptoms—Symptoms, not Diseases, | 333 |

